# Potassæ Chloras in Typhoid Fever

**Published:** 1858-03

**Authors:** V. H. Taliaferro

**Affiliations:** Atlanta, Georgia


### ARTICLE II.

*Potassæ Chloras in Typhoid Fever.* By V. H. TALIAFERRO, M. D., Atlanta, Georgia.

The views which we have been led to entertain in relation to Typhoid Fever, its etiology, pathology, &c., is inconsistent with the plan of treatment as usually applied. Upon many pathological points of the disease, great discrepancy of opinion exists; yet upon its more important features, there seems to be more unanimity of opinion; for instance, that the disease is asthenic in its character; that there exists a disposition to rapid disintegration and disorganization of the vital tissues, as evidenced by fœtor of the breath, sordes, intestinal ulceration, &c., is not denied. Many of the more unfavorable symptoms presented in Typhoid Fever, occur as an effect of the disposition to intestinal ulceration.

With these considerations, and the applicability of Chlorate of Potassa in mucus ulcerations, we have been led to its use in this much dreaded disease; and we are able safely to accord to it a prominence in its treatment, not as yet obtained by us with other remedies; and from our opportunity for the investigation of the disease, having lived for several years in a locality in which it prevailed as an epidemic, we have been enabled to test the efficacy of the more popular plans of treatment as yet recommended.

With the use of Chlorate of Potassa, from the commencement of Typhoid fever, it becomes, by far, more manageable, losing to no inconsiderable degree that uncontrolable disposition to diarrhœa usually attendant upon the disease; to collection of sordes upon the teeth, and fœtor of the breath; and also, to the formation of the dark, heavy, dry incrustations upon the tongue, so indicative of a low asthenic stage of inflammatory or ulcerative action. Its precise therapeutic action we do not pretend to explain, but are satisfied that its beneficial effects are mainly through its alterative action upon the blood. It is far from our intention in the present communication, to enter fully upon the pathological indications of Typhoid Fever, or the therapeutic action of Chlorate of Potassa, but merely to bring to the notice of the profession a remedy which in our hands has been attended with results exceeding by far our expectations. We find our patients treated with the Potassa less subject to diarrhœa, to tympanitis and tenderness cf the abdomen, to less nervous derangement, and rarely delirum.

In stomatits it is an admitted remedy of great efficiency, and in pseudo membranous croup recently recommended by Weatherly of Montgomery as a remedy of very decided power, and also by Brown, of N. C., as a cure for ulceration of the cervix uteri. Thus we see, that in diseases of mucus membranes—whether of the stomach, intestinal canal, the larynx or the uterus, it is a remedy of decided and powerful efficacy. We do not pretend to say that the primary lesion in

Typhoid fever is in the mucus membrane of the intestinal canal, but do say that probably the most troublesome symptoms which occur in the progress of the disease are those resulting from inflammation and ulceration of the intestinal glands. The diarrhœa which results from this glandular ulceration is not unfrequently troublesome in the extreme and if not duly arrested, hurries on the patient to an alarming state of prostration, from which he may not rally, but pass from delirium and collapse, to death. For this diarrhœa Chlorate of Potassa is an appropriate remedy, and relieves it upon the same principle, upon which it relieves, ulceration of the stomach, larynx and uterus.

We usually combine with the Chlorate, Veratrum Viride, which while it does not deteriorate from its alterative properties, adds to it a sedative action upon the circulation, so desirable in Typhoid fever. The two following cases are illustrative of our plan of treatment with the Chlorate of Potassa:

*Case* 1*st.*—Mr. P——, some 22 years of age, was taken on the 20th of January with pain in the head, back and extremities, attended with high febrile excitement; tongue dry, red upon the tips and edges, and but slightly coated, thirst and dryness of the mouth, with some little cough and soreness of the throat. A mild laxative was administered, which operated freely, so much so that it became necessary to check its action with an opiate, after which he was put upon the following

℞—Chloras Potassæ, Sat. Sol., ℥iv.

Tinct. Veratrum Viride, ʒ½

Mix.

Sig.—One tablespoonful every three hours through the day, with an opiate at night.

This constituted the amount of medical treatment through a regular attack of Typhoid Fever. His pulse after the first few days continued from about 90 to 105; his tongue never assumed the dark crusty appearance, usual in typhoid fever. There was no delirium, no subsultus tendinum, no tympanitis, no disposition to diarrhœa. He is now, (Feb. 6,) free of fever and convalescing beautifully, with return of appetite, strength,

&c. Besides the medical treatment, sponging, quantity and quality of diet was strictly enjoined. And in this connection we would remark, that in our humble opinion the treatment of typhoid fever is frequently unsuccessful in consequence of a sufficient amount of nourishment being withheld. While rapid disintegration of vital tissues are going on for three, four or six weeks, unless the wasting system be freely nourished, it must soon be consumed by the combustion resulting from the greatly excited circulation, which with double rapidity is coursing every tissue and calling for renewal of its component elements. We have reason to believe that not unfrequently typhoid patients have perished in consequence of an insufficient amount of nutriment. Wine was also in this case administered from three to four times a day after the first week of his sickness.

*Case 2d.*—Son of Mr. ——, some 12 years of age, was taken to his bed on the 23d of January, after complaining several days of having felt unwell. When called, we found him with febrile excitement very high, pulse beating about 130, with pain in the head, abdomen and extremities. His mind was considerably excited, as was also the nervous system. He complained of severe pains and tenderness of the abdomen, which presented some unusual tightness upon pressure. The tongue presented a heavy yellow coat, with red and dry edges. The symptoms altogether in this case we considered of unusual severity and were apprehensive as to the final issue. Previous to our call a purgative had been administered, which had operated excessively, and very much exhausted the patient. From the commencement of the attack this case presented unmistakable evidences of the nature of the disease. After the administration of an opiate for quieting nervous excitement and allaying irritability of the intestinal canal, he was put upon the following

℞—Chloras Potassæ, Sat. Sol., ℥iv.

Tinct. Veratrum Viride, ʒ½.

Mix.

Sig.—Two teaspoonsful every three hours through the day and night, with an opiate at bed time, to be discontinued if nausea of the stomach should occur, which we never designedly produce in typhoid fever.

In six or seven days from the commencement of treatment his tongue had moistened, and the tips and edges lost its fiery red; nervous excitement had in a great degree subsided; the bowels had lost their tightness and tenderness, with less disposition to diarrhœa; the pulse had reduced to 110. With the continuance of this treatment, with wine, nourishment, sponging, &c., this case went safely and smoothly through his attack, without aggravation of his symptoms to any degree of severity. He is now (Feb. 8) convalescent and discharged.

