# Erratum

**Published:** 1858-04

**Authors:** 


### Erratum.

In March number, page 389, eleventh line, instead of "Anodynes and Sedatives," read Anodynes and Soporifics. And in 15th line, Soporifics and Anodynes, instead of " Sedatives and Anodynes."

